# Usability of Individualized Head-Related Transfer Functions in Virtual Reality: Empirical Study With Perceptual Attributes in Sagittal Plane Sound Localization

**DOI:** 10.2196/17576

**Published:** 2020-09-08

**Authors:** Claudia Jenny, Christoph Reuter

**Affiliations:** 1 Musicological Department University of Vienna Vienna Austria

**Keywords:** head-related transfer function, sound localization, immersive virtual reality, binaural virtual acoustics, 3D audio perception

## Abstract

**Background:**

In order to present virtual sound sources via headphones spatially, head-related transfer functions (HRTFs) can be applied to audio signals. In this so-called binaural virtual acoustics, the spatial perception may be degraded if the HRTFs deviate from the true HRTFs of the listener.

**Objective:**

In this study, participants wearing virtual reality (VR) headsets performed a listening test on the 3D audio perception of virtual audiovisual scenes, thus enabling us to investigate the necessity and influence of the individualization of HRTFs. Two hypotheses were investigated: first, general HRTFs lead to limitations of 3D audio perception in VR and second, the localization model for stationary localization errors is transferable to nonindividualized HRTFs in more complex environments such as VR.

**Methods:**

For the evaluation, 39 subjects rated individualized and nonindividualized HRTFs in an audiovisual virtual scene on the basis of 5 perceptual qualities: localizability, front-back position, externalization, tone color, and realism. The VR listening experiment consisted of 2 tests: in the first test, subjects evaluated their own and the general HRTF from the Massachusetts Institute of Technology Knowles Electronics Manikin for Acoustic Research database and in the second test, their own and 2 other nonindividualized HRTFs from the Acoustics Research Institute HRTF database. For the experiment, 2 subject-specific, nonindividualized HRTFs with a minimal and maximal localization error deviation were selected according to the localization model in sagittal planes.

**Results:**

With the Wilcoxon signed-rank test for the first test, analysis of variance for the second test, and a sample size of 78, the results were significant in all perceptual qualities, except for the front-back position between own and minimal deviant nonindividualized HRTF (*P*=.06).

**Conclusions:**

Both hypotheses have been accepted. Sounds filtered by individualized HRTFs are considered easier to localize, easier to externalize, more natural in timbre, and thus more realistic compared to sounds filtered by nonindividualized HRTFs.

## Introduction

### Theories

The question raised in the article, “Binaural Technique: Do We Need Individual Recordings?” by Møller et al [1], is one that many researchers and developers still ask themselves. The increasing access to advanced virtual and augmented reality technologies gives this topic a particular immediacy. There are different schools of thought as to whether it is important to have personalized head-related transfer functions (HRTFs) for a realistic reproduction of auditory scenes via headphones in virtual reality (VR). The ability to adapt nonindividualized HRTFs via training [2] or the given tolerance by adding distance perception [3], auralization [4,5], auditory motion [6], and cross/multimodal perception [7-10] as well as different recorded auditory stimuli (eg, noise bursts, speech, music) still brings up the question in the title of Møller et al [1]. However, it is well accepted that individualization has a significant effect in sagittal plane sound localization with static target position without visual stimulus [1,11,12]. There are several VR studies where the focus lies on immersive VR, which is helpful in health care (the more immersive the better) [13] or in spatial navigation memory assessment [14], though considerations about immersive audio are missing in these studies. Audio is often neglected in VR studies (eg, [15]), even regarding the sound quality [16], not to mention HRTFs. If and to what extent the perception quality of sound signals in VR can be improved by using individualized HRTFs has not been investigated yet.

### Background

Acoustic localization is the ability to determine and report the position of a virtual sound source and is based on the processing of auditory localization features such as monaural and binaural features [17-21]. It is assumed that binaural and monaural spectral features are processed largely independently of each other [22,23]. While binaural disparities such as interaural time and level differences play an important role in sound localization in the lateral dimension (left-right), monaural spectral cues are known to determine the perceived position of the sound source in the sagittal planes (front-back and up-down). Sound localization in sagittal planes relies on spectral features caused by the filtering effects of the human body [24].

HRTFs describe the acoustic filter effect through the torso, head, and pinna [24-26]. A set of HRTFs (also called “binaural HRTF”) includes the primary localization cues: interaural time differences, interaural level differences, and the monaural spectral cues [24]. This acoustic filter of our own anatomy is individually different and highly frequency-dependent. When HRTFs are measured in the listener’s own ears, it is described as “individual,” “own,” or “listener-specific,” whereas “nonindividual,” “other,” or “generic” HRTF refer to measurements from a different listener, a dummy head, or a calculation from a model.

In order to present virtual sound sources via headphones, the audio signal can be filtered with HRTFs. In this so-called binaural virtual acoustics, the spatial perception may be limited if the used HRTFs deviate from the individualized HRTFs of the listener [11]. This can lead to incorrect virtual sound source positions or even to a localization within the head.

### Prior Work

Individual features should be used to ensure realistic replication, as previous studies have shown that by using listener-specific HRTFs for headphone reproduction, subjects could locate the source of the sound just as accurately as if they were listening to something in free-field reproduction [27,28]. Their research results also showed that subjects with nonindividualized HRTFs have significantly greater localization errors, especially in the median plane, and that front-back confusion increases. However, the results of other studies show that subjects with nonindividualized HRTFs have no localization loss in the horizontal plane with voice stimuli [29] nor do inexperienced subjects acknowledge any significant impact on front-back confusion with individualized HRTFs [30]. Furthermore, studies [31-33] have shown a worsening of externalization or a significant increase in the inside-head localization and an increase in the localization errors in the distance perception in subjects who heard stimuli with nonindividualized HRTFs. Romigh and Simpson [34] confirmed that the replacement of listener-specific interaural features by generic interaural features did no harm but replacing listener-specific monaural features with generic monaural features did interfere with localization performance. Localization models such as the probabilistic model for stationary localization errors in sagittal planes [35] can be used to predict localization errors, which a listener would have had with HRTFs from another listener.

### Goal of This Study

Our study examines the need for individualization of HRTFs in headphone reproduction and the impact of customizability of binaural performance in audiovisual virtual environments. The aim of the study was to find out if and to what extent the perception quality of sound signals in VR can be improved by using individualized HRTFs.

The hypotheses of this experiment can be summarized as follows:

Hypothesis 1: General HRTFs such as the KEMAR (Knowles Electronics Manikin for Acoustic Research) HRTF lead to limitations of 3D audio perception in VR.

Hypothesis 2: The localization model for stationary localization errors is transferable to nonindividualized HRTFs in a multimodal representation.

For the general HRTF, we have chosen the KEMAR HRTF from the Massachusetts Institute of Technology (MIT) KEMAR database [36], which is one of the most widely used HRTFs in both science and industry. The artificial head used to obtain the data has the dimensions of an average human ear and body. We assumed that generic HRTFs lead to limitations of 3D audio perception in VR, such as sound sources would be more difficult to localize and internalize and the tone color would be unnatural, and in general, perceived as unrealistic. However, front-back confusions would be unlikely because listeners were able to move their heads [37]. For the nonindividualized HRTFs, we have chosen HRTFs from the Acoustic Research Institute (ARI) HRTF database. The difference between the KEMAR HRTF and the HRTFs from the ARI database is that the KEMAR HRTF is measured from a dummy head and the HRTFs from the ARI database are measured from human subjects.

## Methods

### General Information

For the study, 39 subjects rated individualized and nonindividualized HRTFs in an audiovisual virtual scene by using a questionnaire, which consisted of 5 perceptual qualities (localizability, front-back position, externalization, tone color, realism; see definitions in Table 1) and was based on the spatial audio quality inventory [38] and the study of Simon et al [39]. A head-mounted display was used to present an acoustically located flying dynamic sound source (drone) in a winter landscape environment. Switching HRTFs took place via touch controllers enabled with the plugin [40]. The filter algorithms took the listener interaction into account in real time. The VR listening experiment consisted of 2 tests: in the first test, subjects rated their own versus a general HRTF (MIT KEMAR dummy head, [36]) and in the second test, their own versus 2 other nonindividualized HRTFs from the ARI HRTF database. As a basis for the selection of nonindividualized HRTFs, the localization model in the sagittal plane by Baumgartner et al [35] was used, which predicts localization errors. For the experiment, 2 listener-specific, nonindividualized HRTFs with a minimum and maximum localization error deviation were selected. For the selection, 140 HRTFs from the ARI database were chosen.

**Table 1:** Perceptual qualities for the assessment of the audiovisual scene derived from the studies on spatial audio quality inventory [38] and that of Simon et al [39].

Perceptual quality	Circumscription	Scale end label
Localizability	If localizability is low, the spatial extent and location of a sound source are difficult to estimate or they appear diffuse. If localizability is high, a sound source is clearly delimited. Low/high localizability is often associated with high/low perceived extent of a sound source [38].	More difficult to easier
Front-back position	Refers to the position of a sound source before or behind the listener only. Impression of a position difference of a sound source caused by “reflecting” its position on the frontal plane going through the listener [38].	Confused / not confused
Externalization	Describes the distinctness with which a sound source is perceived within or outside the head regardless of the distance. Terminologically often enclosed between the phenomena of in-head localization and out-of-head localization [38].	More internalized to more externalized
Tone color bright to dark	Timbral impression, which is determined by the ratio of high-frequency to low-frequency components [38].	Darker to brighter
Realism	Sounds seem to come from real sources located around you [39].	Nonrealistic to realistic

### Subjects

A total of 39 subjects took part in the study. All of them (males, 26/39, 67%; females, 13/39, 33%) had absolute hearing thresholds within the 20-dB range of the average normal hearing population in the frequency range between 0.125 kHz and 12.5 kHz. The 39 subjects had a mean (SD) age of 30.03 (6.738) years (age range, 22-47 years), and about half were experienced listeners (low expertise, 19 subjects; high expertise, 20 subjects). The “low expertise” group included, for instance, lay listeners, who might have been music lovers but were not trained musicians. The “high expertise” group included experienced listeners such as trained musicians, “Tonmeister,” and sound engineers [41]. To determine the required number of subjects, we conducted an a priori power analysis with the software program G*Power (Heinrich Heine University Düsseldorf). A two-sided *t* test with Wilcoxon signed-rank test (one sample case) was assumed, which resulted in a total sample size of 35 with an expected mean effect size of *d*=0.5 for an α error of .05 and a test power of 1–β of 80%.

### HRTF Measurement

HTRFs were obtained for each subject individually by measuring in a semianechoic chamber. The same apparatus and procedure as reported by Majdak et al [42] were used. With a loudspeaker arc of 22 vertically arranged loudspeakers (custom-made with 10 BGS drivers, Vifa), 1550 measuring positions were achieved. The loudspeakers were arranged in the elevation direction from –30° to 80°, with a 5° spacing except between 70° and 80° with a 10° increment. The radius of the loudspeaker bow was 1.2 m. The rotation of the turntable with chair took place in 2.5° increments. For the recording in the ear canal, in-ear microphones (Sennheiser KE-4-211-2) were used. The microphones were connected to the digital audio interface via amplifiers (Radio Design Lamps FP-MP1). An electromagnetic tracking system (Flock of Birds, Ascension) was used to monitor the head position and orientation. As signals during the measurement were exponential, sine sweeps were used with a signal length of about 1.8 seconds, starting at 50 Hz and ending at 20 kHz. To reduce the total time taken to measure HRTFs, we used the multiple exponential sweep method [43]. The HRTF measurement procedure took approximately 60 minutes for each subject, including instruction, reference measurements, and adjustments. The measuring process itself took about 20 minutes.

### Stimuli

**Figure 1:**
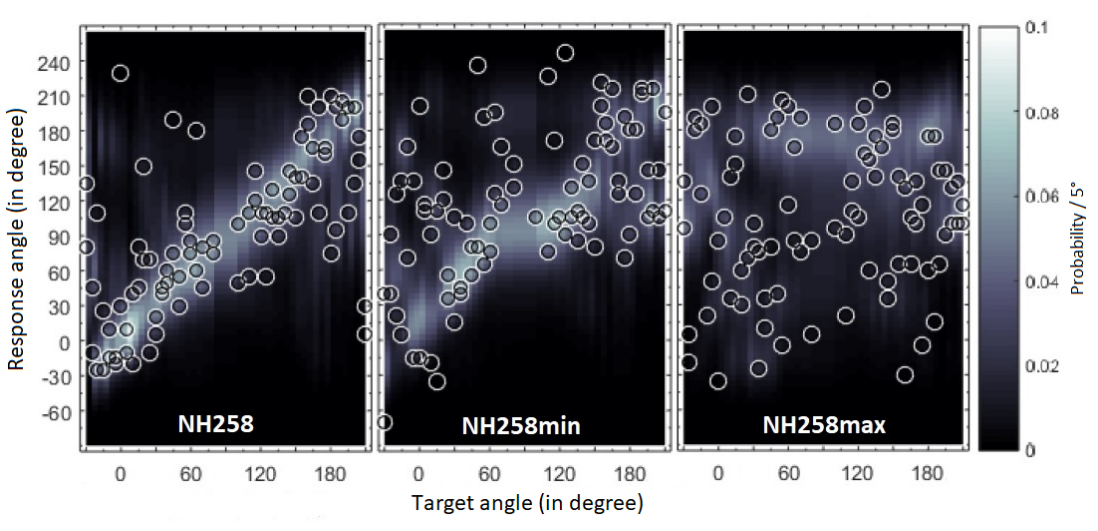
Localization model for prediction of localization errors in the sagittal plane. Probabilistic response predictions are encoded by brightness according to the color bar to the right. Predicted response angles are shown as open circles. NH258: normal hearing listener number 258; min: minimal deviant head-related transfer function; max: maximal deviant head-related transfer function.

As an acoustic stimulus, the synthetically generated stimulus, Gaussian white noise was selected. Gaussian white noise is often used as a stimulus in HRTF studies and was applied to simulate a drone noise. The stimulus was filtered by individualized and nonindividualized HRTFs. The selection of nonindividualized HRTFs in the second test was based on the sagittal plane localization model by Baumgartner et al [35]. This model can predict localization errors of static sound source positions in an auditory-only environment. For the experiment, 2 listener-specific, nonindividualized HRTFs with a minimum and maximum localization error deviation were selected. The deviations of the stationary localization errors were given by means of this model in quadrant error (QE) in percentage and root mean square local polar errors (PEs) in degrees. The QE and PE were calculated using the model via the Auditory Modeling Toolbox in Matlab [44]. Based on the subjects’ own HRTFs (template), it is possible to predict how large QE and PE are when the subjects then hear another HRTF (target). For the range of minimum and maximum HRTFs, 2 conditions were defined to ensure comparability: 1st condition, minimum nonindividualized HRTF range, QE of 10%-30%, PE of 33°-42°, maximum nonindividualized HRTF range, QE of 30%-50%, and PE of 43°-52.25°; 2nd condition, minimum distance between individual/minimal and minimal/maximal HRTFs, QE of 3%, and PE of 3°. An individual, minimal, and a maximal deviant HRTF is shown in Figure 1 using the example of the listener NH258 (normal hearing listener number 258). The individualized HRTF of NH258 had an initial value of QE 18.2% and PE 36.1°. For example, a slightly minimal deviant HRTF would be that of NH157, with which NH258 would have QE of 21.3% and PE of 40.7°. A maximal deviant HRTF would be NH89, with which NH258 would have a QE of 40% and PE of 46.7°. For all HRTFs, the sensitivity parameter was set to the default value 0.5 in the model. All the selected minimal and maximal deviant HRTFs were calculated individually for each subject.

### Apparatus

**Figure 2:**
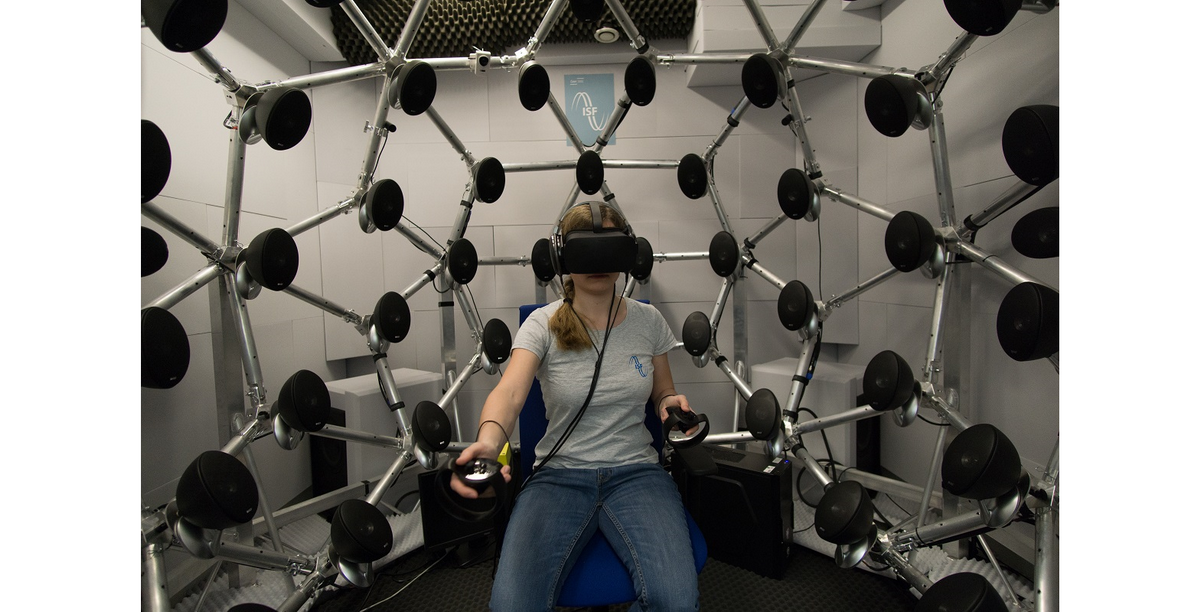
Experimental setup with the Oculus Rift head-mounted display in a semianechoic room. Test environment with open, dynamic, circumaural Sennheiser HD 650 headphones, 3 sensors for the tracking system, and touch controllers for switching the head-related transfer functions. The loudspeaker array was not in use in this experiment.

The virtual acoustic stimuli were presented via headphones (HD 650, Sennheiser) in a semianechoic room. As shown in Figure 2, the listener was seated on a height-adjustable swivel chair in the middle of the room. The virtual visual environment, created with Unity version 2017.3.1f1, was presented via a head-mounted display (Oculus Rift CV1 headset, 2 PenTile organic laser-emitting diode displays, 2160×1200 combined resolution for both eyes, 90 Hz refresh rate, 110° FoV) including touch controller for switching the HRTFs using the Barebone gaming PC in Thermaltake housing with Intel (R) Core (TM) i5-6500 CPU 3.2 GHz processor, 16 GB RAM, 64-bit operating system (Windows 10), 200 GB SSD, GTX 1060 graphics card (6 GB VRAM), HDMI 1.3, 4x USB 3.0, 2x USB 2.0, mouse, keyboard, screen and 3 sensors for head-tracking. Stimuli were generated using the “SOFA (Spatially Oriented Format for Acoustics) Spatializer” plugin [40] and output with a 48-kHz sampling rate filtered with individualized and nonindividualized HRTFs. The “SOFA Spatializer” plugin is a Unity native plugin based on C/C++ for enabling playing HRTF in the SOFA format [45]. The virtual visual environment was created in C# by using the Unity game engine. Three tracking sensors captured the position and orientation of the head in real time. The front 2 sensors were connected with USB 3.0 and the back with USB 2.0. All sensors were fixed at the same height, slightly above the head height. The 2 front sensors had a distance of 1.8 m and the rear sensor had a straight line distance of 2.55 m from the farthest front sensor. The range of motion was 2.4×2.4 m². The sensors were 1.2 m away from the headset. The sensor settings thus corresponded to all specifications for the head-mounted display.

### Description of the Test Environment

The VR listening test took about 60 minutes per subject. The subjects initially were each given an informed consent form and the list of attributes to familiarize themselves with the technical terms (Table 1). Before the actual VR listening test, a pilot study with 3 subjects was conducted to test if the experimental design worked. Two tests were performed.

#### Individualized and General HRTFs

In the first test, the subject was given a rather easy task: the evaluation of 2 HRTFs, that is, their own and the general KEMAR. Both the subject and the experimenter did not know which HRTF (A or B) was assigned (double-blind study). In addition, the set of questions concerning the HRTF was randomized in terms of the subject as well as the repetition. However, each subject was aware that one was the individualized HRTF and the other the general HRTF.

**Figure 3:**
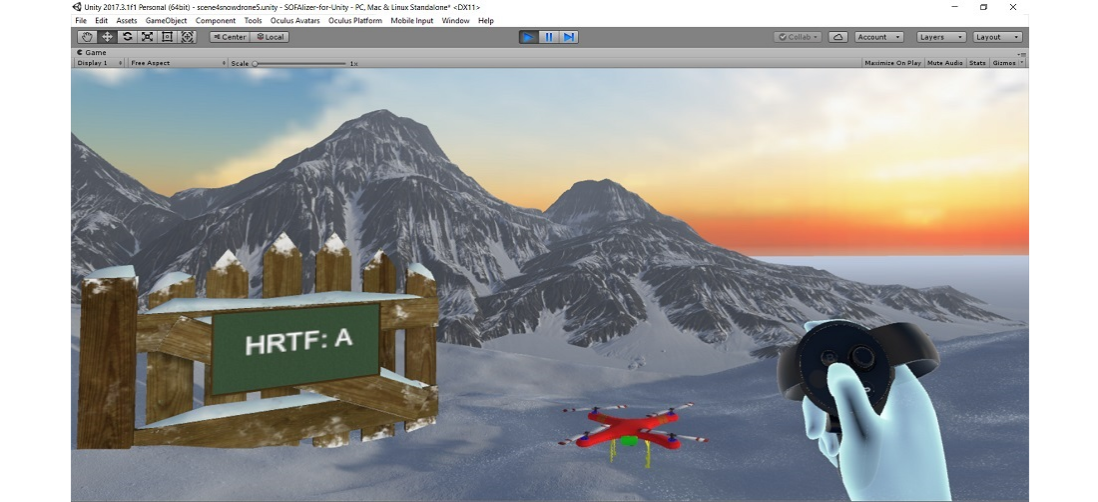
Experimental environment in first-person view: winter landscape scene with drone, board for the display of head-related transfer function (HRTF), and touch controllers for switching.

In the scene, a drone flew overhead along the sagittal planes, landing in front and flying back. In polar coordinates, the audiovisual stimulus flew between –30° and 210° with a distance of 1.2 m (same dimensions as HRTF measurement) back and forth. One animation cycle lasted 24 seconds: 10 seconds for each semicircle flight plus 2 seconds for each landing. A continuous Gaussian white noise was used as the auditory stimulus. The visual stimulus, the drone with 4 rotating rotor blades, served as a guide (visual aid) in which the position of the sound source was supposed to be straight. In addition, haptic touch was added. The avatar hands with the touch controllers were used to switch the HRTFs with a simultaneous display on a fence with a blackboard. Figure 3 shows a screenshot from the subject’s point of view. At the start of the scene, the subject was given about 2 minutes to familiarize herself with the scene and the touch controllers. With the right touch controller, the subject could switch between HRTF A and B via buttons A and B. Sitting on the swivel chair, the subject was allowed explorative movements with her head and body and was not instructed to move her head in any particular way [29,46]. The subject was instructed to explore the VR world by switching between the HRTFs and then rate the HRTFs in the respective perceptual quality. The experimenter was in the same semianechoic room as the subject. The subject was given the tasks by the experimenter, for example, “rate the localizability of HRTF A from 1 to 5 and HRTF B from 1 to 5, with 1 being more difficult to localize and 5 easier to localize.” The subject then explored the VR world and switched between the HRTFs. As in the study by Hendrix and Barfield [47], no time limit was set, but there was a condition to listen to at least one animation cycle of the drone for each perceptual quality. Once the subject was able to rate the HRTFs, the scene was paused, and the HRTFs were scored. The subject’s response was documented by the experimenter. Accordingly, the subject proceeded through the query catalog (Table 1).

#### Individualized and Nonindividualized HRTFs

In the second test, the subject rated 3 HRTFs: HRTF X, HRTF Y, and HRTF Z. One of them was their own HRTF again and the other 2 were HRTFs from other people in the database, one of which was very similar to and the other with large deviations from the individualized HRTF of the subject. The left touch controller was used for switching between HRTF X, Y, and Z. The same procedure was followed as in the first test, rating all the attributes (Table 1). The subject left the glasses on during the scoring; all HRTFs were evaluated simultaneously, and the subject could not return to a different attribute. The duration of each test was also documented. In total, 5 perceptual qualities were tested on 5 HRTFs in 1 repetition, ie, 5×5×2 = 50 answers.

In order to find out whether order effects played any role and to obtain a variance within the subject, we performed a repetition with randomized HRTFs. Before the repetition, there was a break of about 10 minutes in which the subject could take off the glasses. The individualized HRTF was rated twice—once in the first and then in the second test. This served as a reference for checking the functionality of the test design. In addition, after the test, subjects were able to comment on further differences apart from rating the HRTFs.

## Results

### Overview

The evaluation of the attribute localization, externalization, and realism for the first test (individual vs KEMAR) was done with the Wilcoxon signed-rank test and for the second test (individual vs minimal vs maximal) with analysis of variance (ANOVA) and Tukey as ANOVA posthoc analysis. The evaluation of the attributes front-back position was made via the chi-square test with the Fisher test as posthoc and the tone color via the interquartile range.

In order to determine whether different manipulations of stimuli led to different physiological reactions within a group, we applied two-sided *t* tests. Subtests were calculated using *t* tests to investigate possible differences between groups with different expertise and repetition. The groups of different expertise were divided into “low expertise” and “high expertise.” Judgment reliability within the first and the second tests was checked by repetition. There were no significant differences in all perceptual qualities, which meant that with high probability, subjects were able to evaluate all HRTFs reliably (without guessing) with repeated query in spite of randomization. Judgment reliability between the first and second test was assessed by rating the individualized HRTF twice. Evaluation of the individualized HRTF in the first versus the second test showed no significant difference except in externalization, when the *P* value was .04. We attempted to minimize the fatigue effects by the randomized design and a break at halfway through the VR experiment.

Overall, in both tests, statistical significance was found for all perceptual qualities, except in the front-back position between individual and minimal HRTFs. Plots were calculated and created using the statistics program RStudio (RStudio Inc). In the following section, we offer a detailed statistical analysis of the first and the second tests in terms of the tests themselves and the 5 perceptual qualities (scale of 1-5): localizability (more difficult to easier), front-back position (confused to not confused), externalization (more internalized to more externalized), tone color (darker to brighter), and realism (nonrealistic to realistic).

### Individualized and General HRTFs

#### Localizability

**Figure 4:**
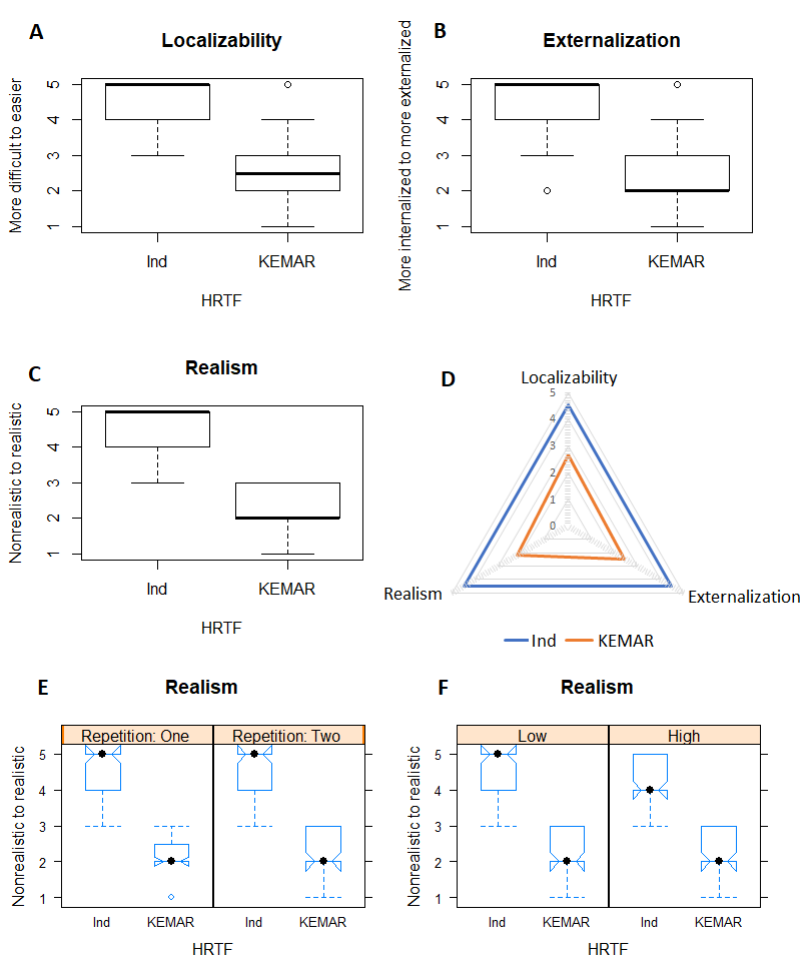
Result of the first test (individual vs KEMAR). A: Localizability overall box plot; B: Externalization overall box plot; C: Realism overall box plot; D: Radar chart for localizability, externalization, and realism; E: Realism repetition response behavior; F: Realism expertise response behavior. Ind: individual HRTF; KEMAR: Knowles Electronics Manikin for Acoustic Research; HRTF: head-related transfer function.

In the assessment of localizability in the first test, the individual HRTF and KEMAR HRTF, which were evaluated with the Wilcoxon signed-rank test, differed significantly (W=5569.5, *P*<.001, Figure 4). The KEMAR HRTF was considered more difficult and the individual HRTF more easily locatable. The subgroup analysis showed great agreement on this result, both between the first and second repetition and in low and high expertise (significance values in Table 2).

**Table 2:** Significance values of the subgroups for localizability, externalization, and realism in the first test.

Perceptual quality	Individual HRTF^a^, *P* value	KEMAR^b^ HRTF, *P* value
Localizability repetition	.46	.76
Localizability expertise	.87	.07
Externalization repetition	.26	.71
Externalization expertise	.60	.87
Realism repetition	.84	.22
Realism expertise	.06	.87

^a^HRTF: head-related transfer function.

^b^KEMAR: Knowles Electronics Manikin for Acoustic Research.

#### Front-Back Position

For the evaluation of the front-back position with its bipolar response scale (“yes” for confused/“no” for not confused), the Pearson’s chi-squared test (a frequency test/independence test) with Yates continuity correction was used. The 39 subjects gave 78 responses in 2 rounds. There was no front-back confusion for the individual HRTF (0%). On the other hand, for the KEMAR HRTF, there were 21 front-back confusions reported for 78 responses (27%). The result of the front-back confusions was significantly different (*χ*^2^_1,77_=22.0, *P*<.001). The KEMAR HRTF was confused by experienced listeners in 14 out of 78 responses (18%) and by inexperienced listeners in 7 out of 78 responses (9%). Due to the possibility of movement by means of head-tracking, basically, no front-back confusion should have occurred [6,37], but the head movements of the subjects were not restricted and therefore could be static as well as dynamic. In practice, movements occur naturally—sometimes more and sometimes less in VR. Nevertheless, with the KEMAR HRTF and a moving stimulus along the sagittal plane, it was still possible that in the VR condition, the visual stimulus was perceived at the front, but the auditory stimulus at the back or vice versa. Wightman and Kistler [37] also detected front-back confusion with uncontrolled sound source movement in their study.

#### Externalization

The results for the perceived externalization of the test items of the KEMAR and individual HRTF with the Wilcoxon signed-rank test were as follows: the individual HRTF was more significantly externalized than the KEMAR HRTF (W=5741.5, *P*<.001, Figure 4). The subgroups showed no significant differences (Table 2). Although the visual stimulus flew over the head, some subjects reported that the auditory stimulus actually flew through their heads and was thus perceived as being more internalized. This was especially the case when the visual stimulus was just behind the head (not in the field of view). Moreover, when the distance from the auditory stimulus to the visual stimulus was too far, it was rated as 1.

#### Tone Color

A rating of 1 or 5 meant that the sound of the stimulus was different from what the subject normally perceives (unnaturally brighter or darker). Here, the subjects were supposed to give appropriate answers to the internal reference. The rating of the tone color was difficult for some subjects without a direct reference. Nevertheless, on average, subjects rated the individual HRTF in tone color with 3, which was defined as natural. The individual HRTF was rated as natural in 62 out of 78 responses (79%) and the KEMAR only once (1%). The KEMAR HRTF was mostly rated with 5 in tone color; thus, it was perceived as unnaturally brighter.

#### Realism

For the final assessment in the first test with the perceptual quality realism, the overall results are shown in Figure 4. The HRTFs were found to be significantly different, with medians of 5 for individual and 2 for KEMAR (W=6030, *P*<.001). There were no significant differences among the subgroups (Table 2).

### Individualized and Nonindividualized HRTFs

#### Localizability

**Figure 5:**
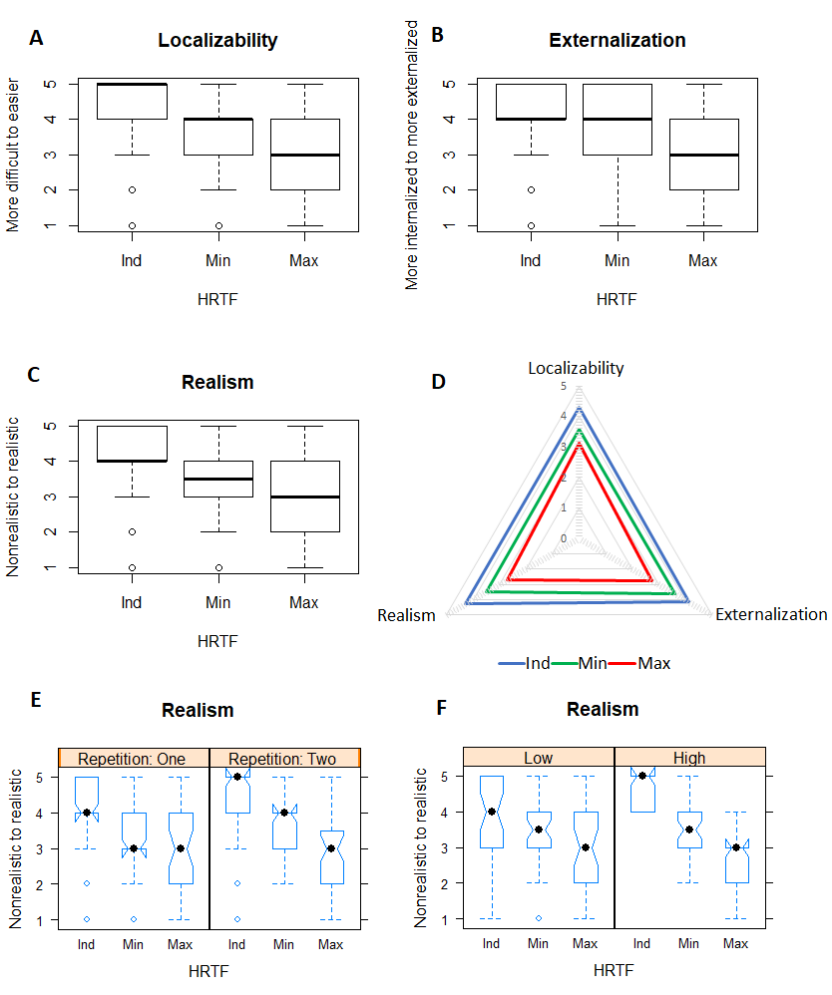
Result of the second test (individual vs minimal vs maximal). A: Localizability overall box plot; B: Externalization overall box plot; C: Realism overall box plot; D: Radar chart for localizability, externalization, and realism; E: Repetition response behavior; F: Expertise response behavior. Ind: individual HRTF; Min: minimal; Max: maximal; HRTF: head-related transfer function.

For the evaluation of the localizability in the second test, an ANOVA with Tukey posthoc test was used to compare the 3 HRTFs. In all HRTF scores, we reached the significance levels (*F*_2,76_=19.131, *P*<.001, Figure 5): individual-maximal (*P*<.001), individual-minimal (*P*=.001), and maximal-minimal (*P*=.049) were calculated using the linear mixed-effects model and the Kenward-Roger method (95% confidence interval). The individual HRTF showed a significantly better behavior than the minimal and maximal HRTFs. In the subgroup, the minimal HRTF was classified as slightly more difficult to localize by the high expertise group (Table 3).

**Table 3:** Significance values of the subgroups for localizability, externalization, and realism in the second test.

Perceptual quality	Individual HRTF^a^, *P* value	Minimal HRTF, *P* value	Maximal HRTF, *P* value
Localizability repetition	.58	.64	.41
Localizability expertise	.06	.03	.32
Externalization repetition	.99	.76	.94
Externalization expertise	.09	.32	.76
Realism repetition	.86	.64	.85
Realism expertise	.04	.78	.29

^a^HRTF: head-related transfer function.

#### Front-Back Position

The evaluation of the significance level of the yes/no results of the front-back position was calculated with the chi-square test and the Fisher test as chi-square posthoc test (*χ*^2^_2,76_=27.0, *P*<.001). There was no significant difference between individual and minimal deviant nonindividualized HRTFs (*P*=.06). However, there were significant differences between individual-maximal (*P*<.001) and maximal-minimal (*P*=.005). The application of the Bonferroni method yielded no changes in the significance level: individual-maximal (*P*<.001), individual-minimal (*P*=.18), and maximal-minimal (*P*=.005). For the individual HRTF again (as in the first test), no front-back confusion occurred (0%). However, the minimal HRTF showed front-back confusion in 5 out of 78 responses (6%) and the maximal HRTF in a total of 19 out of 78 responses (24%). The maximal HRTF was therefore rated significantly worse in the front-back position than the other 2 HRTFs. Listeners with high expertise rated the minimal HRTF in 5 out of 78 responses (6%) as reversed in the front-back position and the maximal HRTF in 14 out of 78 responses (18%). Thus, low-expertise listeners could not find any front-back confusion with the minimal HRTF and they found front-back confusion only occasionally (5/78, 6%) with the maximal HRTF. This result of the subgroup was in agreement with the study by Bronkhorst [30] with regard to inexperienced listeners, for whom hardly any front-back confusion errors occurred.

#### Externalization

For the evaluation of the externalization in the second test, an ANOVA with Tukey posthoc test was used. In all HRTF scores, we achieved significant differences (*F*_2,76_=22.278, *P*<.001, Figure 5): individual-maximal (*P*<.001), individual-minimal (*P*=.04), and maximal-minimal (*P*<.001) were calculated using the linear mixed effects model and the Kenward-Roger method (95% confidence interval). The maximal HRTF showed a significantly worse behavior than the individual and minimal HRTF. The subgroups showed no significant differences (Table 3).

#### Tone Color

In order to statistically analyze the evaluation of the tone color—that is how naturally the respective HRTF was perceived—we used descriptive statistics with the interquartile range [48]. Subjects were unanimous in rating their own HRTF according to the small IQR for their individual HRTF. The IQR was the largest at the maximal HRTF (IQR individual HRTF=0, IQR minimal HRTF=1, and IQR maximal HRTF=2.75), which simply indicated that nonindividualized HRTFs were often perceived as unnaturally brighter or darker as well as unpleasant in timbre. Overall, the individual HRTF was rated as natural in 51 out of 78 responses (65%), the minimal HRTF was rated as natural in 23 out of 78 responses (29%), and the maximal HRTF was rated as natural in 15 out of 78 responses (19%); thus, in some cases, nonindividualized HRTFs sometimes were nevertheless rated as natural in tone color.

#### Realism

All examined HRTFs could be clearly differentiated by rating the attribute realism, of which the overall results are shown in Figure 5. However, the quality of the HRTFs was almost never rated as poor or completely unrealistic. An ANOVA with a Tukey posthoc test showed significant differences in all HRTF scores (*F*_2,76_=31.88, *P*<.001): individual-maximal (*P*<.001), individual-minimal (*P*<.001), and maximal-minimal (*P*<.001) were calculated using the linear mixed effects model and the Kenward-Roger method (95% confidence interval). The subgroup analysis showed that the individual HRTF was rated more often as being more realistic by the more experienced listeners compared to the low expertise group (Table 3).

After the test, subjects were able to comment on further differences apart from rating the HRTFs. With localizability, subjects reported that they had classified HRTFs as more difficult to localize if the auditory stimulus was not congruent to the visual stimulus and was shifted to the right or left or was diffused. Basically, the group of high expertise found it easier to hear differences between the individual, minimal, and maximal HRTFs than the low expertise group. The first test was classified as being easier for some subjects than the second. Many subjects found the VR scene very realistic, but for some, the auditory stimulus was not a realistic sound to match the drone. NH92 and NH785 had difficulty ignoring the artefacts caused by the lack of interpolation and by the error proneness in the HRTF measurement, but in the end, they rated their own HRTF the highest. All HRTFs were equal because none were interpolated. NH794 perceived individual HRTF as much more realistic and its spectrum much closer to reality. None of the subjects experienced motion sickness during the experiment.

## Discussion

### Principal Findings

The most important findings of this study are summarized as follows:

1. In VR, there seems to be a connection between auditory spatialization and the descriptive attribute of realism. The perceived realism increases with the approach to listener-specific spatialization.
2. Significant differences in the evaluation of perceptual qualities in VR seem to be mainly caused by listener-specific features. The presentation with individualized HRTFs in VR shows a greater popularity in the subjective rating than with general or nonindividualized HRTFs.
3. The localization model in sagittal planes based on the stationary pure auditory localization error [35] seems to be transferable to the multimodal audiovisual VR. The subjective evaluation reveals the relevance of localization in the dimension of perceived realism. Even HRTFs with a localization error that only deviates minimally in the static auditory are evaluated as less realistic in a direct comparison with their own HRTFs in a complex scene in a multimodal representation.

### Comparison With Hypotheses

Contrary to our expectations, the use of the tracking system and visual stimuli did not significantly reduce the number of front-back confusions for the KEMAR and maximal HRTF. Furthermore, the results prove the following concerning our hypotheses.

The first hypothesis of our study that general HRTFs lead to limitations of 3D audio perception in VR was confirmed. The first test (individual vs KEMAR HRTF) showed that subjects with a general artificial head HRTF had more difficulties locating moving sound sources in VR. They were confused in the front-back position, found it to be more internalized, and rated the tone color as unnatural and unrealistic. However, the test was performed only with a general HRTF—the KEMAR HRTF from the most widely used MIT KEMAR database [36]—and is thus valid only for this HRTF. In order to make a global statement, several general HRTFs, for instance, that of Neumann KU100, should be included in the investigation. Moreover, the comparison between individual and KEMAR HRTF can be criticized, as the resolution of the individual HRTF was better, with 1550 positions and 256 samples at 48 kHz, compared to the KEMAR HRTF with 710 positions and 512 samples at 44.1 kHz, although the sampling rate had been adjusted. The resolution could therefore be another factor, but it is obvious that the KEMAR HRTF, which has been used almost exclusively in games with spatialization, leads to limitations in 3D audio perception. Although we considered a downgrade of the individual HRTF, no consensus with the same position measurement points could be found.

The second hypothesis on the transferability of the localization model for stationary localization errors for nonindividualized HRTFs in more complex environments such as VR was unequivocally confirmed with the second test of this study. Admittedly, the HRTF selection could have been even more specific to determine the correlation between the localization error and perceived realism in truly complex environments. In the future, the deviations of the stationary localization errors could be represented by means of the model in percentage: for instance, individualized HRTFs would have a localization error deviation of 0% and nonindividualized HRTFs with increasing inaccuracy at an increasing value of up to 100% (=error at random localization). Thus, more than 2 nonindividualized HRTFs could be selected at fixed percentages and a finer resolution of the degree of realism compared to the localization error would be possible. Moreover, other databases could have been included, but we wanted to maintain the comparability of the measurements. Finally, only 2 nonindividualized HRTFs were selected for the second test because the number of HRTFs in the database was too small to make general statements.

### Comparison With Prior Work

Studies such as those of Begault et al, Hendrix and Barfield, and Larsson et al [29,47,49] have already tried to examine the relationship between realism and improving spatialization with HRTF rendering, but they found no significant differences. This is probably due to the lack of understanding or the unclear definition of what is meant by the assessment of realism. The explanation by Hendrix and Barfield [47] for their findings was that the subjects might have interpreted the realism in terms of the visual realism “scene realism” and not the overall quality of the performance. Additionally, Begault et al [29] argued that no differences in realism were found, because subjects probably had no common understanding of what the perceived realism implied. Furthermore, Larsson et al [49] did not define the queried realism in advance and suggested that the subjects had made the auditive realism more dependent on well-designed source content (eg, a bus really sounds like a bus) instead of on one accurate 3D performance (that the bus is properly externalized and located). In our study, the concept of realism was defined in advance according to the study by Simon et al [39]. Thus, a common understanding of the queried realism was guaranteed for all listeners.

By examining the differences in the perceptions between individualized and general or nonindividualized HRTFs in VR, reproduction systems are to be examined in order to generate virtual and augmented realities as realistically as possible. Unlike in the study by Berger et al [50], in this study, limitations in 3D audio perception with general HRTFs in VR arose. It is questionable whether this claim in the title of the study carried out by Microsoft Research with the MIT KEMAR HRTF dataset alone is justified without comparison to individualized HRTFs. Further, it should be noted that the Berger study did not evaluate elevation but only azimuth.

Another point is the learnability or the adaptability of foreign HRTFs. Studies [2] have shown that HRTFs can be learned and adapted through training in a short amount of time, sometimes even within minutes [51], but this has only been evaluated by localization performance. Whether an adaptation in the evaluation of externalization, tone color, or realism in VR is possible remains a question. Presumably, learning new HRTFs comprehensively is a longer process and more akin to learning a foreign language.

Current research especially for serious games in VR and mental health often mention that “The literature suggests that immersion is largely influenced by both visual and audio qualities” [52], but audio is rarely a topic in such studies. “VR excels in its advantage of being able to draw on both audio and interactive visual stimuli, making the fearful stimuli appear as real as possible [53],” while by using personalized HRTFs, the stimuli would be even more present, immersive, and realistic.

### Limitations

In a realistic virtual scene, the reverberation should not be neglected. This could be set in the reference setup via the plugin (Plugin Spatializer Reverb in the Audio Mixer, [40]) as well as via the Unity Engine (Audio Reverb Filter, Reverb Preset). However, the reverberation leads to another variable with many parameters. In our study, all effects were therefore examined in free field. This may sound unrealistic but this corresponds to a real situation in a room that is acoustically dry. For further experiments, the reverberation can be integrated as an additional variable building on this work*.*

### Conclusions

Both hypotheses have been accepted: first, general HRTFs lead to limitations of 3D audio perception in VR and second, the localization model for stationary localization errors is transferable to nonindividualized HRTFs in more complex environments such as VR. The results of the first test (individual vs KEMAR HRTF) and of the second test (individual vs minimal vs maximal) show that sounds filtered by individualized HRTFs are considered easier to localize, easier to externalize, more natural in timbre, and thus more realistic compared to sounds filtered by nonindividualized HRTFs. In conclusion, the most realistic simulation of sound sources in virtual environments can be achieved by using individualized HRTFs, which leads to an improvement in terms of the following perceptual qualities: localizability, front-back position, externalization, tone color, and realism. Therefore, future VR studies, especially in serious games, should take an auditory spatialization with individual HRTFs in their experiments into account.

To answer the question “Binaural Technique: Do We Need Individual Recordings?” by Møller et al [1] in the field of VR, this study provides empirical evidence. The answer is in the affirmative. Listener-specific filtering in headphone reproduction helps achieve a truly realistic 3D audio perception in VR. In order to see the topic of the necessity of a higher realism content in VR by means of individual HRTFs, less from theoretical basic research and more from the side of practical realization, the following example provides a nice vivid comparison: HRTFs are like a suit. It fits you perfectly when it is tailor-made.

## Abbreviations

ANOVA: analysis of variance
ARI: Acoustic Research Institute
HRTF: head-related transfer function
KEMAR: Knowles Electronics Manikin for Acoustic Research
MIT: Massachusetts Institute of Technology
NH: normal hearing listener
PE: polar error
QE: quadrant error
SOFA: Spatially Oriented Format for Acoustics
VR: virtual reality
